# Biological fundamental functional units of meridians: mechanosensitive ion channels

**DOI:** 10.3389/fmed.2026.1775475

**Published:** 2026-05-26

**Authors:** Yiming Wang, Xiaojing Zhou, Songbin He

**Affiliations:** Department of Neurology, Zhoushan Hospital, Wenzhou Medical University, Zhoushan, China

**Keywords:** acupuncture, functional units, mechanosensitive ion channels, meridians, Traditional Chinese Medicine

## Abstract

Meridians are central to the theory of Traditional Chinese Medicine (TCM), yet their biological essence remains elusive. Mechanosensitive ion channels (MS channels) respond to diverse physical stimuli, and their properties closely parallel the biophysical characteristics attributed to meridians. Here, we propose the hypothesis that MS channels serve as the fundamental biological functional units of meridians. To test this hypothesis, we systematically compared the properties of MS channels with those of meridians and utilized Zusanli (ST36) as a model to dissect the critical role of MS channels in mediating the complete signaling pathway of acupuncture—from local initiation and central integration to visceral effector responses. Furthermore, applying this hypothesis successfully addresses three major challenges in meridian research: (1) the efficacy of sham acupuncture, which arises from the ubiquitous distribution of MS channels and their response gradients; (2) variations in the pathways of propagated sensation along meridians, which result from MS channel activity being guided by anatomical networks and modulated by central processes; and, importantly, (3) the persistence of meridian function following organ transplantation, which can be coherently explained by a mechanism wherein systemic signals from the recipient remotely regulate MS channels on the cells of the transplanted organ, thereby providing a modern biological basis for the concept of “meridian transplantation.” This hypothesis provides a testable molecular framework for understanding the essence of meridians, bridging traditional functional descriptions with modern developmental biology, neuroscience, and cellular and molecular mechanisms.

## 1 Introduction

Meridians are a functional system established in ancient Chinese medicine, serving as a cornerstone of Traditional Chinese Medicine (TCM) theory. Although the intrinsic nature of meridians remains a subject of ongoing investigation, their clinical applications—particularly therapies such as acupuncture and moxibustion—have garnered widespread recognition. Currently, acupuncture and moxibustion are practiced in 193 countries and regions worldwide, providing therapeutic relief to millions of patients.

In 1980, the World Health Organization (WHO) formally endorsed acupuncture and moxibustion as valuable therapeutic interventions. In October of that same year, the WHO Regional Office for the Western Pacific established a standardized international nomenclature for acupuncture point locations (1). Since then, extensive clinical research on acupuncture therapies has been conducted (2), with numerous high-quality randomized controlled trials demonstrating their efficacy in treating a diverse array of diseases (3).

However, because the intrinsic biological nature of meridians remains unclear, research designs and efficacy evaluations often suffer from methodological flaws and misinterpretations, leading to numerous negative reports regarding these therapies. Many clinical trials have failed to detect significant differences between true acupuncture or moxibustion and sham procedures (4, 5). Furthermore, the use of these therapies for hypertension has been criticized for lacking robust evidence of efficacy, with some researchers even concluding that they hold no therapeutic value for this condition (6).

Therefore, there is an urgent need to elucidate the intrinsic nature of meridians and the mechanisms underlying acupuncture and moxibustion to resolve these ongoing controversies. In recent years, numerous researchers, including those from China, have undertaken in-depth explorations into this issue and proposed various hypotheses (7, 8). These include the neural, immunological (9), super-anatomy (10), fascial (11), and Merkel cell hypotheses (12, 13). Unfortunately, none of the proposed hypotheses have comprehensively addressed all questions related to meridians, particularly concerning their physiological and pathological effects on effector cells. Consequently, elucidating the intrinsic nature of meridians remains a century-old challenge in the field of TCM.

## 2 Meridians and mechanosensitive ion channels

According to TCM theory, meridians are pathways through which Qi and blood circulate, connecting the internal organs (viscera) with the body surface and regulating bodily functions. In TCM, Qi is the fundamental substance that constitutes the human body and sustains vital activities; it also serves as a driving force for physiological functions. It represents both a refined material foundation and a manifestation of vital activities. Qi circulates within the meridians, is distributed throughout the body via acupoints, regulates visceral functions, and maintains the balance between the internal and external environments. Acupoints are specific sites on the body surface where the Qi of the meridians is infused; they serve as the sites of action for therapies such as acupuncture and massage. Meridians are traditionally regarded as functionally interconnected networks of acupoints. Stimulating acupoints on the same meridian tends to elicit similar physiological effects on the corresponding internal organs. According to TCM, meridians perform four primary physiological functions, which can be summarized as follows:

(1) Communication and Connection: The human body is organized at multiple structural levels—comprising cells, tissues, organs, and systems—all working in concert to sustain life. As the fundamental units of life, cells aggregate to form the four basic tissue types (epithelial, connective, muscle, and nervous), which further integrate into organs and systems. Each organ possesses distinct physiological functions; however, it is through the connecting role of the meridians that these activities are harmonized and integrated, enabling the body to operate as a cohesive whole.
(2) Circulation of Qi and Blood to Nourish Tissues and Organs: TCM posits that Qi and blood are the fundamental substances underpinning vital activities. Meridians serve as the pathways through which Qi and blood circulate to warm, nourish, and sustain all parts of the body, including organs, tissues, and other structures, thereby maintaining their normal physiological functions.
(3) Sensation and Conduction: Meridians are responsible for sensing stimuli and transmitting information. When a specific area of the body is stimulated, the meridians convey this signal internally to the corresponding organs, eliciting relevant physiological or pathological responses. These internal changes, in turn, may manifest externally on the body surface via the meridian system.
(4) Regulation of Visceral Functions: The meridian system helps regulate and balance the functional activities of the human body. Should dysfunction occur in a specific organ, therapeutic interventions such as acupuncture can be employed to stimulate the regulatory capacity of the meridians, thereby facilitating the restoration of normal physiological functions.

In summary, meridians serve as a functional system that influences physiological and pathological processes at both the cellular and organismal levels, primarily through the circulation of Qi and blood.

Chinese scholars have conducted extensive research on the biological characteristics of meridians. Studies have demonstrated that mechanical (14), electrical (15), acoustic (16), optical (17), magnetic (18), and thermal (19) stimulation of acupoints along meridians can induce a range of biological effects with physiological and pathological significance. These effects are not localized merely to the stimulation site but also extend to distant corresponding organs, as well as the nervous and humoral systems.

Historically, meridian research faced skepticism due to the absence of identifiable structural units capable of sensing diverse stimuli, including electrical, acoustic, optical, magnetic, thermal, and mechanical signals. However, in 1979, Bennett and Clusin identified mechanisms of electroreception that share features with mechanosensitive acoustico-lateralis receptors (20). In 1986, Grigg provided a comprehensive biophysical analysis of mechanoreceptors (21). Subsequently, ion channels responsive to light (22), magnetic fields (23), and ultrasound (24) were discovered. More recent advances in the study of temperature and tactile receptors have highlighted the critical role of mechanosensitive ion channels (MS channels) in biological processes (25, 26), offering new perspectives for understanding the mechanisms of acupuncture and moxibustion within the meridian system.

## 3 Hypothesis based on MS channels

The development of an organism originates from the continuous differentiation of a single cell—the fertilized egg. If meridians are inherent functional systems of living organisms, then their fundamental functional units should exist from the very beginning of life. A meticulous analysis of protein functions on the membrane of the fertilized egg reveals the presence of MS channels across various species (27). Studies have confirmed that such channels exist across all three domains of life: Eukarya, Bacteria, and Archaea. These MS channels play crucial roles in the formation, development, and maturation of organisms (28, 29). Upon mechanical stimulation, the influx of cations into the cytoplasm mediates a diverse array of cellular responses, including cell growth, migration, adhesion, morphogenesis, gene expression, fluid homeostasis, and vesicle transport (30). Although bacterial MS channels primarily function as osmolyte release valves, their evolutionary conservation and related structural studies provide profound insights into the fundamental principles of mechanosensation, which may enhance our understanding of mammalian systems.

**FIGURE 1:**
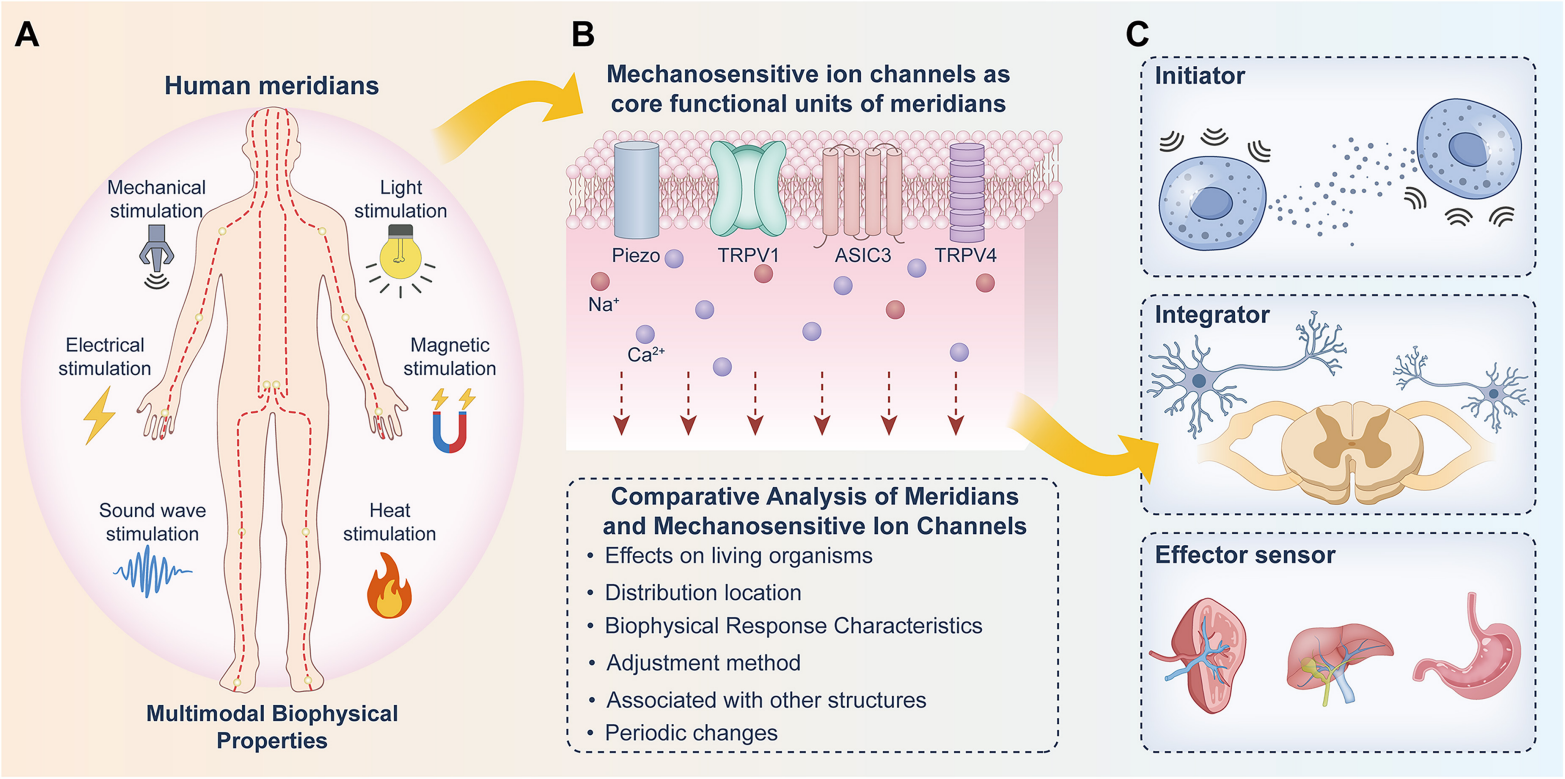
Schematic diagram illustrating the hypothesis that mechanosensitive ion channels serve as the fundamental biological functional units of meridians. **(A)** The meridians of the human body possess various biological characteristics. **(B)** MS channels exhibit similarities to meridians across six biological characteristics. **(C)** MS channels play a critical role in mediating the complete signaling pathway of acupuncture and moxibustion, spanning local initiation, central integration, and visceral effector responses.

From an evolutionary perspective, it is plausible that as organisms evolved from single-celled to multicellular and embryonic forms, they developed tissue structures and systems capable of efficient mechanical force transduction to harness the biological role of mechanical stimuli. Extensive multidisciplinary research has demonstrated that anatomical structures at the organ, tissue, and cellular levels are as critical to mechanotransduction as individual mechanosensitive proteins. The human body employs hierarchical structures—systems within systems—comprising interconnected networks that span from macroscopic to nanoscopic scales, thereby concentrating stress on specific mechanosensitive molecules (31). Studies have shown that cytoskeletal contraction forces, which drive the convergence and extension of cell populations, play a key role in processes such as gut formation in *Xenopus laevis* embryos and wound healing (32). Numerous structures and signaling molecules, including stretch-activated ion channels, caveolae, integrins, cadherins, growth factor receptors, myosin motors, cytoskeletal filaments, nuclei, and extracellular matrix components, have been implicated in mechanotransduction pathways (31). The remarkable functional and evolutionary links among ion channels, active membrane transporters (pumps), and even certain water-soluble proteins may suggest a common evolutionary origin (33). Central to these processes is the transduction of mechanical forces into downstream biological effects. In particular, MS channels convert diverse physical stimuli into electrochemical signals, representing a pivotal node in governing biological responses. Based on this evidence, we propose the following hypothesis: MS channels serve as the fundamental biological functional units of meridians. More precisely stated, MS channels are the key molecular sensors and signal transducers at the cellular level upon which the meridian system relies to execute its biological functions (such as sensation and conduction, central integration, and visceral regulation). They are the essential molecular components underpinning the complex functions of meridians, and their roles are realized by embedding within and modulating macroscopic systems, such as the nervous system, fascia, and humoral pathways, as shown in Figure 1.

This perspective is strongly supported by developmental biology. Studies have demonstrated that MS channels (e.g., Piezo1) are highly expressed in embryonic stem cells, and their activity evolves during the differentiation process. They serve as key molecules that enable stem cells to respond to mechanical cues and determine their differentiation fate (34). At higher tissue levels, the mechanical properties of the extracellular matrix guide the differentiation of mesenchymal stem cells into neurons, myoblasts, or osteoblasts via MS channels. More importantly, during organ morphogenesis, MS channels (e.g., Piezo1) precisely guide the migration pathways of cells, such as neural crest cells (35). These findings indicate that MS channels, serving as fundamental mechanosensors, are deeply involved in the entire embryonic developmental program—from cell fate determination and tissue differentiation to organ morphogenesis. Therefore, the “fundamental functional unit” in our hypothesis exists not only in mature individuals but is also rooted in the very origin of life development, potentially establishing the primitive molecular and cellular foundation for the innate, specific functional connections between meridians and viscera.

We propose that MS channels serve as the fundamental biological functional units of meridians, yet they do not constitute the meridians themselves. Meridians are now widely recognized as complex systems comprising multiple interconnected components (36–38). However, without the involvement of MS channels, the principal biological functions of meridians would be difficult to achieve. Although numerous proteins and signaling pathways contribute to the therapeutic mechanisms of acupuncture and moxibustion, MS channels play a particularly critical role. Current evidence indicates that these channels influence a diverse array of proteins and signaling cascades. For instance, Piezo channels transduce mechanical signals that trigger diverse downstream responses during cellular differentiation, including the activation of the integrin, ERK1/2 MAPK, Notch, and WNT signaling pathways (39).

Numerous studies have investigated the influence of meridians on biological effects across various cell types; however, earlier research seldom addressed the role of MS channels. In recent years, as the understanding of these channels has deepened, a growing body of evidence has highlighted their significant contributions to the biological effects associated with meridians. Functional coupling (40) and crosstalk (41) occur not only among different types of MS channels but also between these channels and other proteins. Abnormally hyperactive MS channels are detrimental to normal physiological functions. Studies have revealed abnormally high activity of MS channels in tumor cells (42), with distinct MS channels playing specific roles in tumor growth and migration (43, 44). Mechanosensing—mediated by Piezo1, adhesions, Yes-associated protein (YAP), and transcriptional coactivator with PDZ-binding motif (TAZ)—influences tumor–immune interactions and the efficacy of immunotherapy (45). These findings suggest that within the highly complex physiological and pathological networks composed of neurohumoral components, MS channels act as integrators.

## 4 Comparison of biological characteristics between meridians and MS channels

**FIGURE 2:**
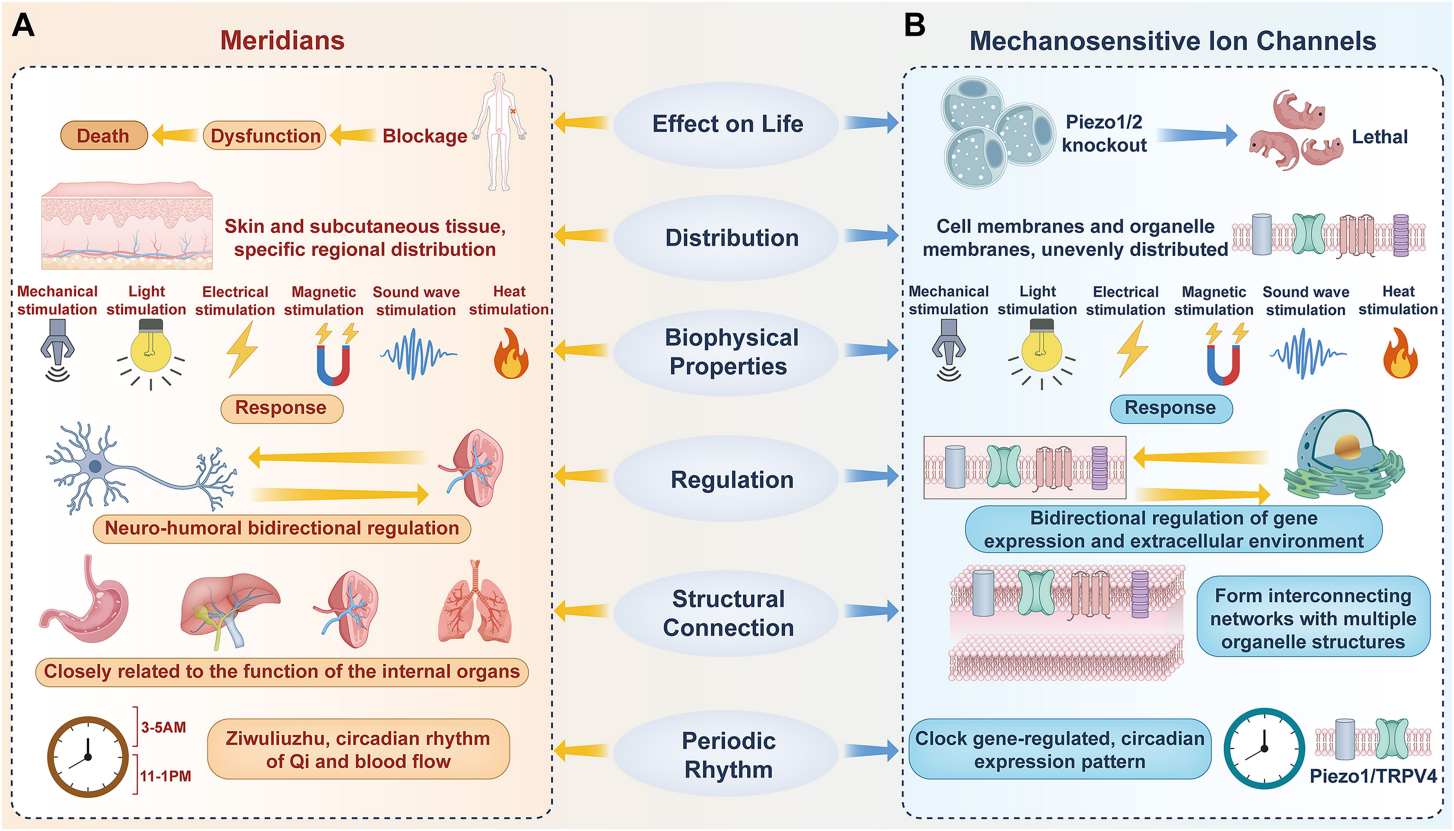
Mechanosensitive ion channels share similarities with meridians across six biological characteristics. Effects on Life: **(A)** When meridians are obstructed or their functions are lost, physiological processes of the organism become disrupted, potentially leading to death. **(B)** Knockout of the *Piezo1/2* genes can result in embryonic lethality. Distribution: **(A)** Meridians are distributed within specific areas of the skin and subcutaneous tissues. **(B)** MS channels form helical arrays on the cell body, exhibiting a non-uniform distribution. Biophysical Properties: **(A)** Meridians are reactive to mechanical, acoustic, electrical, optical, magnetic, thermal, and other stimuli. **(B)** MS channels can respond to diverse stimuli, including mechanical, electrical, acoustic, optical, magnetic, and thermal signals. Regulation: **(A)** Meridians play a regulatory role within the nervous and humoral systems. Conversely, the functional states of the nervous and humoral systems also influence the excitation states of meridians and acupoints. **(B)** The functions of MS channels are regulated not only by cellular genes but are also influenced by both intracellular and extracellular environments. Structural Connection: **(A)** The functional states of internal organs are closely related to specific meridians. **(B)** Extensive interconnectivity exists between MS channels and other key cellular and subcellular structures. Periodic Rhythm: **(A)** The circulation of Qi and blood within the meridians follows a specific circadian rhythm, commonly referred to as Ziwuliuzhu. **(B)** The circadian expression of MS channels is correlated with the expression levels of *Clock* genes.

Meridians are present within the skin and subcutaneous tissues of the body, whereas MS channels are localized within the cytoplasmic membrane. These represent two distinct levels of biological organization. A single MS channel measures approximately 10 nm in size, while the width of a meridian is approximately 1 mm, representing a difference of five orders of magnitude. Furthermore, meridians are regarded as functional systems rather than discrete anatomical structures, which contrasts with the molecular and hierarchical nature of MS channels. To date, five major classes of MS channels have been identified: epithelial sodium channels/degenerin channels (ENaC/DEG), acid-sensing ion channels (ASICs), two-pore domain potassium channels (K2Ps), transient receptor potential channels (TRPs), and Piezo channels. A growing body of research suggests that MS channels exhibit biological characteristics closely resembling those attributed to meridians. While some of these features show clear correlations, others require further investigation, as summarized in Table 1 and illustrated in Figure 2.

**TABLE 1:** Comparison of biological characteristics between meridians and mechanosensitive ion channels.

Areas of comparison	Meridian	Mechanosensitive ion channels (MS channels)
Effect on life	Present in all living organisms, maintaining their normal physiological functions	Present in all living cells and organisms, maintaining their normal physiological functions (62)
Distribution	Distributed within specific areas of the skin and subcutaneous tissues	Located at specific sites on the cell membrane, rather than being randomly or uniformly distributed (50, 51)
Biophysical properties	Responsive to mechanical, electrical, acoustic, optical, magnetic, and thermal stimuli	Responsive to mechanical, electrical, acoustic, optical, magnetic, and thermal stimuli (24)
Regulation	Regulated by the nervous and humoral systems	Regulated by nuclear genes (62, 63) as well as the intracellular and extracellular environments (64)
Structural connection	Closely associated with visceral functions	Expressed on intracellular membranes, such as the nuclear membrane and endoplasmic reticulum (41, 71)
Periodic rhythm	The circulation of Qi within meridians exhibits a specific temporal regularity	The expression of Piezo1 and TRPV4 channels is correlated with cellular circadian rhythms (79, 80)

### 4.1 Impact on the organism

According to TCM theory, meridians play a vital role in determining life and death; when meridians become blocked or cease to function, the physiological processes of the organism are disrupted, potentially leading to death. Recent studies have demonstrated that MS channels exhibit a similar degree of biological significance. For instance, the knockout of the *Piezo1* gene in mice results in embryonic lethality (46), whereas the knockout of the *Piezo2* gene leads to death within 24 h of birth (47). Furthermore, MS channels have been proven to play a critical role throughout embryonic development (27). Research indicates that all cells express MS channels (48). Therefore, in terms of their essentiality for survival, the role fulfilled by MS channels is comparable to the functions attributed to meridians.

### 4.2 Distribution

According to TCM theory, meridians are distributed across specific regions of the skin and subcutaneous tissues. Based on their functional attributes, these are classified into 12 regular meridians, eight extraordinary meridians, and others. Stimulation of specific acupoints along these meridians is believed to elicit distinct physiological responses. For instance, Taichong (LR3), an acupoint belonging to the Liver Meridian, is situated on the dorsum of the foot in the depression proximal to the junction between the first and second metatarsal bones. Similarly, Neiting (ST44), which pertains to the Stomach Meridian, is located on the dorsum of the foot between the second and third toes, at the dermal transition zone (red and white skin) of the interdigital web. Although these two acupoints are anatomically adjacent, functional magnetic resonance imaging studies have revealed both overlapping and distinct patterns of brain activation in response to their stimulation. Acupuncture at both LR3 and ST44 elicits activation in common regions, including the contralateral primary somatosensory area and the ipsilateral cerebellum. However, stimulation at LR3 specifically activates the contralateral occipital gyrus, ipsilateral medial frontal gyrus, parietal lobe, temporal lobe, rostral anterior cingulate cortex, lentiform nucleus, insula, and contralateral thalamus. In contrast, ST44 selectively activates the ipsilateral secondary somatosensory area, contralateral middle frontal gyrus, inferior frontal gyrus, lingual gyrus, lentiform nucleus, and bilateral posterior cingulate cortex (49). Notably, MS channels—proteins predominantly located on the plasma membrane of cells and bacterial bodies—exhibit a distribution pattern that parallels the anatomical specificity of meridians. Research has identified large- and small-conductance MS channel proteins in specific regions of bacterial extracellular membranes. Their distribution is non-uniform, reminiscent of the spatial organization of bacterial cytoskeletal proteins. These channels have been rigorously shown to form helical arrays on the cell body, suggesting functional compartmentalization (50, 51). Therefore, both MS channels and meridians share a common feature: they are not randomly or uniformly distributed on the surface of organisms.

### 4.3 Biophysical properties

In recent years, numerous studies have revealed that meridians possess the following biophysical properties:

(1) Responsiveness to mechanical stimuli: Manual acupuncture at acupoints can elicit the transmission of meridian sensation and induce physiological effects (14).
(2) Responsiveness to sound waves: Sound waves can propagate along meridians and acupoints, with a conduction velocity faster than that in soft tissue but slower than that in bone (52).
(3) Sensitivity to electrical stimulation: Acupoints exhibit lower electrical resistance compared to surrounding tissues (53). Combined electrical and mechanical stimulation can trace a sensory pathway consistent with classical meridian trajectories (54).
(4) Sensitivity to light: The optical characteristics of human meridians differ from those of adjacent tissues; for instance, light attenuation along the Pericardium Meridian is lower than that in non-meridian pathways (55).
(5) Sensitivity to magnetic stimulation: Applying linear magnets with different magnetic pole orientations leads to significant changes in electroencephalogram (EEG) activity in treatment groups targeting the Heart Meridian (56).
(6) Sensitivity to thermal stimulation: Moxibustion-induced heat transfer along the Heart and Lung Meridians is typically more pronounced at distal points of the corresponding meridians than at proximal points of unrelated meridians (57).

Notably, MS channels located on the surface of the cell membrane respond to a diverse array of stimuli, including mechanical, electrical, acoustic, optical, magnetic, and thermal signals (detailed in Table 2). This broad responsiveness helps explain the diverse biophysical properties observed in meridians and serves as a key supporting element for the hypothesis proposed in this study.

**TABLE 2:** The biophysical characteristics of meridians and the response mechanisms of mechanosensitive ion channels.

Biophysical characteristics of meridians	Response mechanism of mechanosensitive ion channels	Experimental evidence
Responsiveness to mechanical stimuli	Mechanical stimulation activates MS channels (25, 26)	Manual acupuncture at acupoints elicits propagated sensation along meridians and physiological effects (14)
Reactivity to acoustic waves	Ultrasonic stimulation activates MS channels (24)	Acoustic waves propagate through meridians and acupoints with a conduction velocity faster than that in soft tissues but slower than that in bones (52)
Sensitivity to electrical stimulation	Individual electrical pulse stimulation of Piezo1 channels increases the synthesis of metabolic signals and proteins in muscle cells (120)	Electrical resistance at acupoints is significantly lower than that of surrounding tissues (53)
Sensitivity to optical stimulation	Photothermal effects induce changes in membrane tension, leading to channel activation (22)	The optical properties of human meridians differ from those of adjacent tissues; optical attenuation along the Pericardium Meridian is lower than that in non-meridian regions (55)
Sensitivity to magnetic stimulation	Magnetic fields alter the orderliness of membrane lipids, thereby stimulating mechanical gating (23)	Significant changes in EEG activity were observed when targeting the Heart Meridian using linear magnets with different magnetic pole orientations (56)
Sensitivity to thermal stimulation	TRPV channels are thermally activated, inducing an increase in Ca^2+^ influx and generating excitatory signals (121, 122)	Moxibustion-induced heat transfer between the Heart and Lung Meridians is typically more pronounced at distal points of the corresponding meridians than at proximal points of unrelated meridians (57)

### 4.4 Regulation of brain neural and cellular mechanotransduction via acupoint meridians

Functional imaging studies have demonstrated that stimulating acupoints along meridians can activate specific regions of the human brain. Acupoints located on the same meridian tend to elicit activation in overlapping or functionally related brain areas. Moreover, clinically frequently used acupoints appear to exert more significant modulatory effects on cortical regions compared to less commonly used acupoints (58). For instance, acupuncture at Neiguan (PC6) has been observed to enhance activity in the left parahippocampal gyrus, fusiform gyrus, and right superior temporal gyrus, while suppressing activity in the right middle frontal gyrus, right anterior cingulate cortex, and cingulate gyrus. Acupuncture also influences serum hormone levels, leading to increased estradiol alongside decreased luteinizing hormone and follicle-stimulating hormone (59). These findings suggest that meridians play a regulatory role within both the nervous and humoral systems. Conversely, the functional states of the neural and humoral systems can also affect the electrical excitability of meridians and acupoints. Additionally, the catecholaminergic system is primarily involved in the electroacupuncture-mediated regulation of cellular immunity, whereas the serotonergic system plays a major role in modulating humoral immunity through electroacupuncture (60). Furthermore, acupuncture therapy has been demonstrated to improve chronic cough in post-operative guinea pigs by regulating the transient receptor potential vanilloid 1 (TRPV1) signaling pathway via protein kinase A and protein kinase C (61).

The functions of MS channels are regulated not only by cellular genes (62, 63) but also by both the intracellular and extracellular environments (64). Studies have demonstrated that mechanical forces sensed at the cell membrane interface are converted into intracellular signals, thereby triggering a cascade of downstream events. In certain cases, these events can lead to alterations in gene regulation and protein expression. Through this mechanism, cells can progressively remodel their cortical cytoskeleton and cell membrane, enabling them to adapt to their mechanical microenvironment until they achieve mechanical homeostasis within new stress limits (65). Changes in cholesterol levels have been found to influence both the mechanosensitivity and basal channel activity of MS channels (66). Furthermore, the functional state of these channels can affect gene expression within the cell body and induce changes in the intracellular and extracellular milieus. Research indicates that nuclear mechanics, morphology, and function can be modulated by MS channels, which facilitate cellular perception of the extracellular environment. This process can lead to subsequent alterations in histone modifications and chromatin-based nuclear rigidity (67). For example, in atherosclerosis, transient receptor potential vanilloid 4 (TRPV4) has been demonstrated to reduce the expression of miR-146a in macrophages within aortic tissue (68). Thus, from the perspective of resgulatory mechanisms, MS channels exhibit functional similarities with the concept of meridians.

### 4.5 Phenomena related to other structures

According to TCM theory, the functional states of internal organs are closely associated with specific meridians. Stimulating acupoints along these meridians can modulate the physiological activities of the corresponding visceral organs. For instance, experimental studies have demonstrated that electroacupuncture stimulation at the PC6 acupoint, which belongs to the Pericardium Meridian, reduces the protein and RNA expression levels of ASIC2 and ASIC3 in rats with myocardial ischemia. This intervention also inhibits the opening of ASICs, thereby mitigating damage to cardiomyocytes (69). Similarly, Zusanli (ST36) and Shangjuxu, two acupoints located on the Stomach Meridian, also participate in the modulation of visceral sensitivity. Electroacupuncture stimulation at these acupoints suppresses yeast polysaccharide-induced colorectal hypersensitivity. This protective effect may involve ion channel activity, the downregulation of TRPV1 expression in the colorectum, and the inhibition of the ERK1/2 MAPK pathway in both the peripheral and central nervous systems (70).

In recent years, studies have revealed that MS channels are not exclusively localized to the cell membrane; they are also present on the nuclear membrane, endoplasmic reticulum (45, 71), and other intracellular structures, a finding of significant research value. This distribution pattern suggests that MS channels exhibit extensive interconnectivity with other critical cellular and subcellular structures, a mechanism of action similar to that of the meridian system.

### 4.6 Periodic rhythm

According to TCM theory, the circulation of Qi within meridians follows a specific circadian rhythm, a phenomenon commonly referred to as “meridian flow.” Studies indicate that the anti-arrhythmic effects of the PC6 acupoint may be mediated through the regulation of circadian rhythms (72). Compared to control groups, combining regular acupoint massage with timed acupoint massage, which is guided by the meridian flow theory and considers the specific active periods of meridians, yielded better outcomes in treating systemic headaches than either approach alone (73). Furthermore, acupuncture applied according to the meridian flow method has been demonstrated to alleviate hypothalamic neuronal damage and modulate inflammatory responses in rat models of insomnia. This therapy also upregulates the mRNA expression of *Clock* and *Bmal1* and increases melatonin levels (74).

The first member of the TRP superfamily of MS channels was identified as a protein involved in phototransduction in fruit flies (75). Studies have demonstrated that both temperature and light regulate circadian rhythms through mechanisms involving TRP channels and clock genes (76, 77). Furthermore, TRP channels participate in a systemic neural sensory network that coordinates the interaction between sleep and circadian regulation (78). Research in mouse primary cultured urothelial cells has revealed that the circadian expression of Piezo1, TRPV4, Connexin26, and VNUT is correlated with the expression levels of clock genes (79). Additionally, bladder filling may exhibit a circadian rhythm, driven by rhythmic functional changes in Piezo1 and TRPV4 under the control of clock genes (80). These findings suggest that the activity of MS channels follows a distinct temporal pattern, analogous to the periodic electrical excitation observed along meridians. Whether these two types of temporal regularity are correlated warrants further investigation.

## 5 Hypothesis verification: supporting evidence from the ST36 paradigm

With regard to the biological functions of meridians, current research primarily focuses on the efficacy and mechanisms of acupuncture and moxibustion. Although the therapeutic effects and mechanisms of acupuncture and moxibustion cannot be equated directly with the biological functions of the meridians themselves, they can still reflect certain characteristics of the meridian system from a specific perspective. Furthermore, although animal models, particularly anesthetized or genetically modified mice, cannot fully replicate subjective human components such as the “deqi” sensation and psychological expectations, the fundamental mechanisms based on ion channels and neural circuits discovered in these animals likely constitute the common basis for the effects of acupuncture in humans. Studies indicate that MS channels serve as important structures through which acupuncture and moxibustion exert their biological effects (14). Acupoints, acting as key nodes in meridian-based induction and conduction, are typically stimulated by needling or thermal energy that penetrates the skin, passing through the epidermis, dermis, subcutaneous tissue, and muscular layers. While acupoints in different anatomical regions vary in their precise anatomical structures, a common feature is the presence of specific cell populations within these areas. Various acupuncture and moxibustion techniques can elicit characteristic sensations, such as distending pain, numbness, and heaviness. The functional heterogeneity of sensory nerves underlying these experiences depends on the composition and combined activity of ion channels across different cell types (81).

**FIGURE 3:**
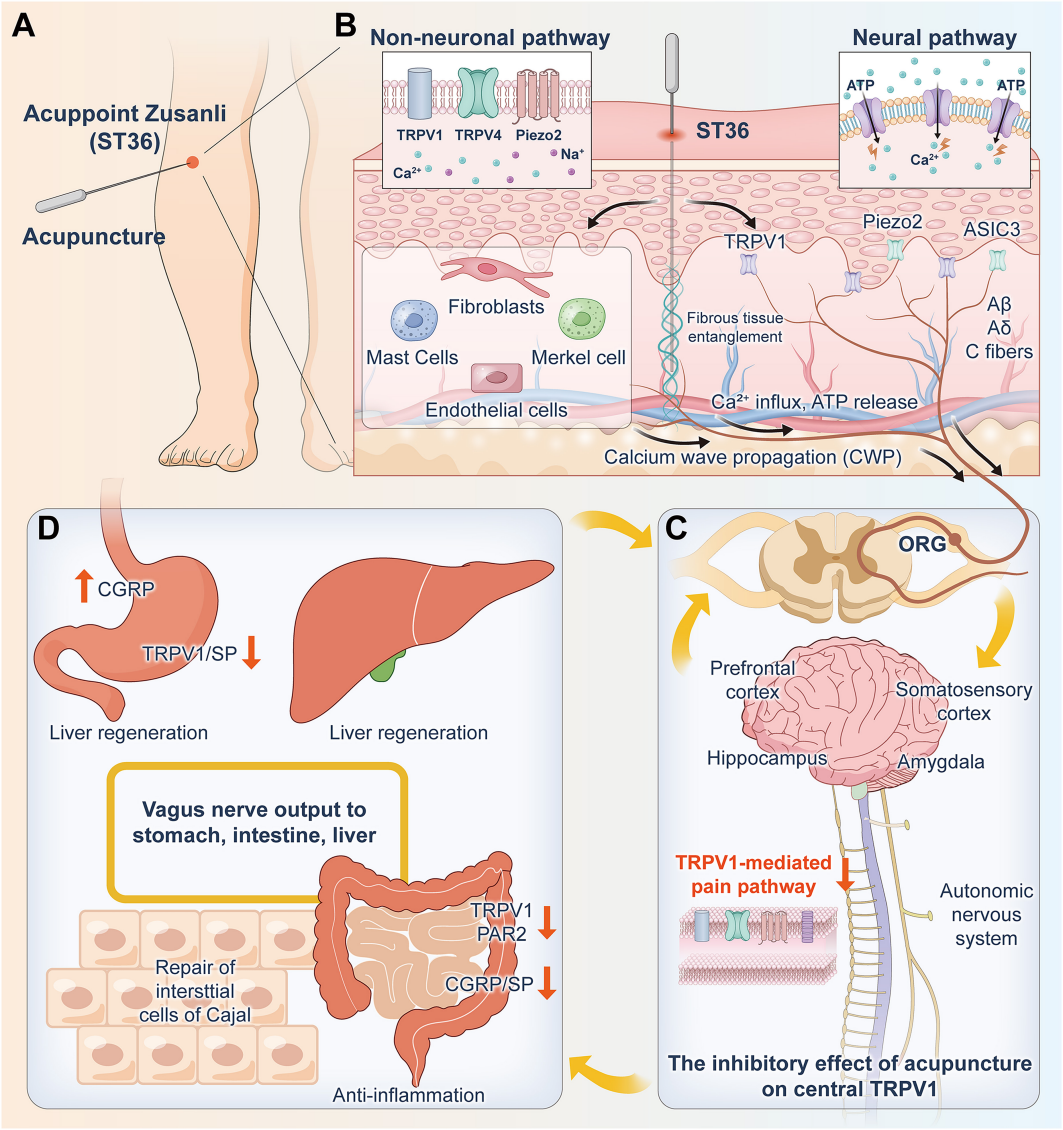
The effects of ST36 stimulation on MS channels across different cellular levels. **(A)** The ST36 acupoint, targeted by acupuncture and moxibustion, is located at the proximal anterolateral aspect of the lower leg. **(B)** High expression levels of TRPV1, TRPV2, Piezo2, and ASIC3 are observed in both neuronal and non-neuronal cells across various anatomical layers of the acupoint, which include the subcutaneous connective tissue, muscles, and nerve fascicles. The manipulative actions of acupuncture and moxibustion, such as needle rotation, entangle fibers and stimulate adjacent structures, including mast cells, fibroblasts, Merkel cells, basal keratinocytes, endothelial cells, free nerve endings, Meissner corpuscles, Pacinian corpuscles, and sweat glands. Stimulating the MS channels within these structures induces the influx of cations, predominantly Ca^2+^, triggers ATP release, and generates calcium wave propagation (CWP). The afferent signals are predominantly transmitted via Aβ, Aδ, and C fibers, with the cell bodies of these neurons residing in the dorsal root ganglion (DRG). **(C)** Acupuncture signals are transmitted and integrated along the classical neuroanatomical pathway, progressing sequentially from the DRG or trigeminal ganglion to the spinal cord or brainstem, thalamus, cortex or limbic system, hypothalamus, brainstem autonomic centers, and ultimately to peripheral effectors. Serving as key molecular modulators, MS channels are widely distributed across multiple nodes of this pathway, and their functional states directly influence signal processing efficiency and the ultimate physiological output. **(D)** Neural signals originating from the central nervous system can regulate the activity or expression of MS channels on these visceral cells either directly or indirectly via the humoral system, thereby altering cellular functions. Furthermore, the inherent mechanical activities of internal organs, such as gastrointestinal peristalsis, generate feedback signals through the MS channels located on their cells. By acting as local mechanoreceptors, these channels further fine-tune the functional states of the organs. This process ultimately achieves the peripheral effector outputs of meridians, specifically the regulation of visceral functions and the circulation of Qi and blood.

In recent years, acupuncture and moxibustion research has focused on numerous acupoints, among which Zusanli (ST36) has received the greatest attention. Located on the proximal anterolateral aspect of the lower leg, ST36 evokes both superficial sensations arising from cutaneous stimulation and deep sensations originating from muscular or tendinous structures. Afferent signaling is conveyed principally by Aβ, Aδ, and C fibers, whose cell bodies reside in the dorsal root ganglion (DRG). Within these neurons, nociceptive stimuli are transduced into electrical signals via the expression of specific ion channels, such as TRPV1 and Nav1.8, and neuropeptides. From a contemporary neurobiological perspective, mechanical stimulation or moxibustion at ST36 would be expected to elicit only localized tissue damage and nociceptive input. Nevertheless, clinical observations consistently demonstrate that such stimuli produce significant physiological and pathological modulations throughout the body (82). Using ST36 as a model, the present study investigates the contribution of MS channels to the therapeutic effects of acupuncture and moxibustion, as illustrated in Figure 3.

### 5.1 Role in local acupoint cells

Recent investigations have confirmed the high expression of TRPV1, TRPV4, and ASIC3 in both neuronal and non-neuronal cells across the distinct anatomical layers of the mouse ST36 acupoint, which include subcutaneous connective tissue, muscle, and nerve fascicles. Compared to non-acupoint gluteal muscle, TRPV1 and ASIC3 are significantly upregulated in muscle tissue and the epineurium, whereas TRPV4 shows preferential enrichment in the epineurium and loose subcutaneous connective tissue (83). All three channels are transmembrane proteins that, upon mechanical stimulation, permit the influx of cations, predominantly Na^+^ and Ca^2+^, thereby depolarizing the cell membrane. In sensory neurons, this depolarization is sufficient to trigger action potentials. Gadolinium, a non-selective MS channel blocker that also inhibits TRPV1, TRPV4, and ASIC3, markedly suppresses mechanically evoked currents in isolated neurons. Consistent with this observation, systemic gadolinium administration prior to manual acupuncture abolishes acupuncture-induced effects in rats (84). The genetic deletion of the *TRPV1*, transient receptor potential vanilloid 2 (*TRPV2*), or *ASIC3* genes attenuates acupuncture-induced analgesia in mice (85). Notably, *TRPV2*-knockout animals exhibit impaired mast cell activation at acupoints and a corresponding reduction in analgesic efficacy compared to wild-type littermates (86). Collectively, these findings demonstrate that local TRPV1, TRPV2, and ASIC3 at acupoints are indispensable for the initiation of acupuncture responses. Further electrophysiological studies reveal that low-intensity electrical stimulation produces analgesia via ASIC3 receptors localized to Aβ fibers, whereas high-intensity stimulation recruits TRPV1 receptors expressed on Aδ and C fibers to exert a systemic antinociceptive effect (87).

Acupuncture at the ST36 acupoint markedly upregulates TRPV1 expression on local nerve fibers. Subcutaneous administration of a TRPV1 antagonist at this acupoint attenuates the anti-inflammatory effects of acupuncture in septic mice (88). Subsequent mechanistic studies demonstrate that TRPV1 within the ST36 region is co-expressed with the calcium wave propagation (CWP) machinery, which includes pannexin-1, connexin-43, P2Y1, and P2Y2 (82). CWP constitutes a feed-forward cascade in which TRPV1-mediated Ca^2+^ influx triggers ATP release; this release subsequently drives further Ca^2+^ waves via P2Y purinoceptors, thereby propagating the sensory signal downstream following TRPV1 activation. Mechanical deformation imposed by acupuncture engages TRPV1 through two convergent pathways. First, in the neuronal pathway, needle manipulation activates TRPV1 on peripheral nerve fibers, evoking a Ca^2+^ influx that directly triggers action potentials or induces ATP release, which subsequently acts on P2X/P2Y autoreceptors to reinforce electrical signaling. Second, in the non-neuronal pathway, TRPV1 expressed on muscle fibers and fibroblasts is activated by mechanical stress, leading to Ca^2+^ entry and ATP efflux. Extracellular ATP then initiates CWP, relaying the acupuncture signal to adjacent nociceptive terminals and onward to the central nervous system (89). Additionally, some studies have utilized acoustic shear waves to activate cellular calcium. These acoustic shear waves employ the anisotropy of tissue elasticity as their waveguide for transmission, and this cellular activation does not rely on the nervous system (90). Parallel evidence demonstrates that mast cell degranulation evoked by mechanical stress, heat, or red light is mediated by a TRPV2-dependent Ca^2+^ influx (91). Collectively, these findings establish that TRPV1 and TRPV2 orchestrate local mechanosensory transduction at the ST36 acupoint, integrating neuronal and non-neuronal components to propagate acupuncture-induced signals systemically (83).

Additionally, the skin at acupoints contains a heterogeneous population of mechanosensory complexes, which include free nerve endings, Merkel cells that mediate sustained touch, Meissner corpuscles that respond to light touch, and Pacinian corpuscles that detect deep pressure and vibration. These structures are located at varying depths; for instance, Meissner corpuscles are superficially situated within the dermal papillae, whereas Pacinian corpuscles are deeply embedded within the dermis or hypodermis. The depth of needle insertion, typically ranging from 1 to 3 cm at the ST36 acupoint, likely engages both superficial and deep mechanoreceptors, alongside muscle spindles and interstitial afferents. Some researchers propose that acupuncture meridians do not represent abstract channels for Qi, but rather constitute an intrinsic neural conduction network within the skin based on C-type nerve fibers and Merkel cells. Furthermore, acupuncture achieves analgesia by blocking signal transmission within this network, and referred itch serves as a physiological manifestation of its activity (92, 93). Recent studies have detected Piezo2 immunoreactivity in Merkel cells, the axons of Meissner corpuscles, and various non-neural tissues such as basal keratinocytes, endothelial cells, and sweat glands (94). Notably, many important and extensively studied acupoints are located in areas of the body surface exhibiting high tactile sensitivity. This observation is consistent with their anatomical features, which include high-density mechanoreceptors and afferent nerve endings. MS channels serve as the key molecular basis enabling these receptors to achieve high-sensitivity mechanotransduction. Consequently, this provides a novel perspective on the role of MS channels within acupuncture meridians, suggesting that the contribution of various mechanosensory complexes to the conduction network of the meridian system represents a crucial area for future research.

### 5.2 Role in central nervous system cells

When acupuncture and moxibustion signals are transmitted from the periphery to the central nervous system, they can inhibit TRPV1-dominated neural pathways across multiple brain regions, which include the prefrontal cortex, somatosensory cortex, hippocampus, hypothalamus, and cerebellum, as well as the spinal cord. This inhibition induces Ca^2+^/calpain-mediated ablation of axonal terminals, reduces the intensity of central synaptic transmission, and attenuates the central sensitization of pain signals (82). The comorbid state of chronic pain and depression induced by saline injection is associated with aberrant TRPV1 expression in cerebellar lobules VI, VII, and VIII. Electroacupuncture at the ST36 acupoint can modulate TRPV1 and related molecular pathways, thereby ameliorating these pathological changes (95). Electroacupuncture therapy also suppresses TRPV1 overexpression within the central nervous system in a mouse model of fibromyalgia (96). Under stress conditions, enhanced activity of the High Mobility Group Box 1 protein(HMGB1), S100 calcium-binding protein B (S100B), and TRPV1 pathways is observed in the prefrontal cortex, somatosensory cortex, thalamus, and amygdala of mice. This effect is attenuated by electroacupuncture at the ST36 acupoint and is similarly diminished in TRPV1^–/–^ mice (97). Furthermore, growing evidence suggests that TRPV1 serves as a key responsive channel for acupuncture manipulation within both the peripheral and central nervous systems (98). Notably, both electroacupuncture and manual acupuncture modulate the expression and function of MS channels, such as TRPV1, within the central nervous system, which may constitute one of their common central analgesic mechanisms.

The regulation of visceral effector cells via meridians plays a crucial role within the autonomic nervous system (ANS) (99). Notably, MS channels are equally essential in modulating the activity of visceral effector cells within the ANS. Excitatory TRPV1 and TRPA1 channels, alongside inhibitory cannabinoid type 1 (CB1) receptors, are functionally expressed along the sensory fibers of the vagus nerve, where they exert both physiological and pathological effects on axonal membrane excitability and neuropeptide release (100). Furthermore, studies have identified the subunits of epithelial sodium channels/degenerin channels as structural components of MS channels in vagal afferents and pressure-sensitive neurons (101).

Research has demonstrated that vagotomy inhibits liver regeneration following partial hepatectomy, thereby confirming the essential role of the vagus nerve in this regenerative process (102). Notably, electroacupuncture at the ST36 acupoint acutely activates cholinergic neurons within the dorsal motor nucleus of the vagus nerve (DMV), leading to enhanced acetylcholine release from hepatic vagal nerve endings. This process subsequently activates interleukin-6 signaling within hepatic macrophages, thereby promoting hepatocyte proliferation and facilitating liver regeneration (103). Furthermore, studies have indicated that vagus nerve discharge induced by ventromedial hypothalamic injury may trigger hepatocyte apoptosis through the Fas/Fas ligand system, a process mediated by the cholinergic pathway in the rat liver (104). Additionally, pretreatment with electroacupuncture at the ST36 acupoint has been reported to reduce oxidative stress and alleviate hepatic ischemia/reperfusion injury in mice via the activation of the vagus nerve-dependent Nrf2 signaling pathway (105).

Moreover, studies have demonstrated that the vagus nerve, which innervates both the airway and abdominal tissues, expresses TRPV1 and TRPA1 to mediate sensory inputs. These depolarizing, calcium-permeable ion channels play a pivotal role in detecting environmental stimuli and endogenous metabolites, thereby inducing neuropeptide release and contributing to neurogenic inflammation (100). Notably, electroacupuncture therapy at the ST36 acupoint has been proven to modulate neurotransmitter dynamics in colitis and nociceptive hypersensitivity. This effect may involve the L6 dorsal root ganglia (DRG), where mediators such as substance P (SP), bradykinin, and prostacyclin (PGI2) participate in suppressing the activation of the TRPV1/CGRP pathway (106).

In summary, acupuncture signals are conducted and integrated along the classical neuroanatomical pathway (DRG/trigeminal ganglion → spinal cord/brainstem → thalamus → cortex/limbic system → hypothalamus → brainstem autonomic centers → peripheral effectors). Serving as key molecular modulators, MS channels are widely distributed across multiple nodes of this pathway, and their functional states directly influence the efficiency of signal processing and the ultimate physiological output.

### 5.3 Role in visceral effector cells

In earlier studies, investigations into the biological effects of acupuncture and moxibustion primarily focused on neurohumoral regulation and microcirculation, whereas relatively limited attention was devoted to their influence on visceral effector cells. Recent evidence, however, has highlighted the important role of MS channels in mediating the regulation of visceral effector cells by acupuncture and moxibustion. For example, the development of acute gastric mucosal lesions has been demonstrated to involve a marked increase in the expression of TRPV1 and SP within the DRG, as well as elevated TRPV1, SP, and malondialdehyde activities in gastric tissues, which are accompanied by a significant decrease in the levels of Calcitonin Gene-Related Peptide(CGRP), nitric oxide, and superoxide dismutase. Pretreatment with electroacupuncture at the ST36 acupoint protects against water immersion-induced gastric mucosal injury by downregulating TRPV1 and SP expression in the DRG and gastric mucosa, while concurrently upregulating CGRP and nitric oxide activity (107). Furthermore, electroacupuncture at the ST36 acupoint has been reported to restore the normal ultrastructure of interstitial cells of Cajal within the gastric antrum and small intestine, resulting in a significantly increased expression of the gap junction protein connexin 43 compared to model groups (108). Moreover, electroacupuncture at the ST36 acupoint can modulate gastric mucosal mediators and intestinal endocrine factors, which include gastrin, serotonin, CGRP, insulin, and pancreatic polypeptide, thereby contributing to the regulation of digestive function (109).

Under conditions of visceral hypersensitivity, the expression of protease-activated receptor 2 (PAR2) and TRPV1 proteins is upregulated, leading to the release of neuropeptides such as SP and CGRP, which are involved in the transmission of nociceptive signals. Studies indicate that electroacupuncture at the ST36 acupoint can alleviate visceral hypersensitivity by inhibiting the expression of nerve growth factor and its receptor NTRK1 via mast cell-mediated peripheral afferent pathways. This intervention also reduces the expression of PAR2 and TRPV1 proteins in gastric tissues and suppresses the abnormal release of neurotransmitters, thereby contributing to the amelioration of hypersensitivity symptoms (110).

Moxibustion has been demonstrated to reduce visceral hypersensitivity in the colonic tissues of mice with chronic ulcerative colitis, thereby alleviating inflammatory infiltration and fibrotic damage. These protective effects appear to be associated with the downregulation of tumor necrosis factor-α (TNF-α), TNF receptor 1, p38 mitogen-activated protein kinase (p38 MAPK), and TRPV1 expression in colonic tissues. Furthermore, studies have confirmed an intensity-response relationship between temperature and gastric motility under local moxibustion-like stimulation, a process in which TRPV1 receptors play a pivotal role in mediating the regulation of gastric motility.

In summary, neural signals from the central nervous system can regulate the activity or expression of MS channels on these visceral cells either directly or indirectly via the humoral system, thereby altering cellular functions. The mechanical activities of the visceral organs themselves, such as gastrointestinal peristalsis, generate feedback signals through MS channels on their cells that act as local mechanoreceptors, which further fine-tunes their functional states. This process ultimately realizes the peripheral effector outputs of meridians, specifically the regulation of visceral functions and the circulation of Qi and blood.

Collectively, these findings derived from the ST36 acupoint demonstrate that MS channels act as local initiators at the acupoint, integrators within the nervous system, and effect sensors within visceral organs. This multilevel involvement provides a coherent, channel-centric mechanistic narrative explaining how a local physical stimulus applied at a specific site can produce systemic therapeutic effects. Consequently, this strongly supports the hypothesis that MS channels serve as the fundamental functional components underpinning meridian-related phenomena.

### 5.4 The broad spectrum of physiological and therapeutic effects of ST36 stimulation: from phenomena to mechanisms

Beyond the roles described above, the literature also details other effects of the ST36 acupoint. To provide a relatively comprehensive reflection of this broad range of physiological and therapeutic effects, we summarize the principal effects of ST36 stimulation validated in human studies and animal models (82, 111), categorized by physiological systems in Table 3.

**TABLE 3:** Effects of acupuncture at ST36 in disease models across different physiological systems.

Physiological system	Pathological conditions ameliorated	Observed effects
Respiratory system diseases	Acute lung injury (ALI), asthma, chronic obstructive pulmonary disease (COPD), acute respiratory distress syndrome (ARDS)	Reduced pulmonary inflammatory infiltration; decreased pro-inflammatory cytokines (IL-6, TNF-α, IL-1β) in bronchoalveolar lavage fluid (BALF) and plasma; improved oxidative stress markers (reduced ROS, MDA; increased GPX4, FTH1); regulated airway mucus secretion (MUC5AC); improved pulmonary function parameters (airway resistance, lung compliance)
Nervous system diseases	Vascular dementia (VD), cerebral infarction, Alzheimer’s disease (AD), autism spectrum disorder (ASD), multiple sclerosis (MS), neuropathic pain	Improved spatial learning, memory, and cognitive functions; reduced central nervous system (CNS) inflammation (inhibited microglial/astrocyte activation; reduced IL-1β, TNF-α, IL-6 in spinal cord/brain); alleviated oxidative stress (modulated Nrf2, NLRP3 inflammasome); regulated hippocampal neuronal activity (increased *c-Fos* expression) and synaptic plasticity (activated cAMP/PKA/CREB pathway)
Digestive system diseases	Intestinal ischemia/reperfusion (I/R), ulcerative colitis (UC), functional diarrhea (FD), gastric pain, chronic atrophic gastritis (CAG), diabetic gastroparesis (DGP)	Reduced gastrointestinal mucosal inflammation and injury; regulated gastrointestinal motility (bidirectional modulation of gastric movement, improved rhythm disorders); restored interstitial cells of Cajal (ICC) networks; modulated gut microbiota composition; alleviated visceral hypersensitivity (reduced colonic 5-HT, gastric PAR2/TRPV1 expression)
Endocrine system diseases	Obesity, type 2 diabetes mellitus (T2DM), insulin resistance (IR), steroid-induced insulin resistance	Reduced body weight and fat accumulation; ameliorated chronic low-grade inflammation (reduced macrophage infiltration in adipose tissue); improved glucose metabolism disorders (lowered blood glucose and free fatty acids; enhanced insulin sensitivity; promoted GLUT4 translocation); improved diabetic complications (e.g., gastroparesis)
Immune system diseases	Sepsis, experimental autoimmune encephalomyelitis (EAE), allergic contact dermatitis (ACD), rheumatoid arthritis (RA)	Regulated immune homeostasis (modulated proportions of T lymphocyte subsets, e.g., Th1/Th2, Treg/Th17); inhibited lymphocyte apoptosis; inhibited mast cell degranulation; reduced systemic inflammatory cytokine storms
Others	Inflammatory, neuropathic, and fibromyalgia pain; anxiety-like behaviors; hypothalamic-pituitary-adrenal (HPA) axis overactivation	Increased pain thresholds; improved anxiety-like behaviors; inhibited HPA axis overactivation; initiated local events at the acupoint, including mast cell recruitment, ATP release, and calcium wave propagation (CWP)

Although stimulating the ST36 acupoint can produce a wide array of physiological and therapeutic effects, clinical studies have demonstrated that different meridian acupoints exhibit functional specificity. For example, acupuncture at acupoints such as Shenmen (HT7), Quze (PC3), and Neiguan (PC6) on the Heart Meridian and Pericardium Meridian has demonstrated substantial therapeutic efficacy in treating arrhythmias and improving myocardial strain. Conversely, the effect of needling the ST36 acupoint on the Stomach Meridian is relatively poor in this regard.

MS channels are widely distributed not only in neuronal and non-neuronal cells within acupoint regions but also in central neurons and humoral-related cells involved in the regulation of acupuncture. Furthermore, a substantial number of these channels are present within visceral cells, enabling these cells to respond to acupuncture stimulation and generate corresponding biological effects. MS channels perform similar molecular functions across different locations and cell types of the meridian system, specifically converting mechanical stimuli or changes in mechanical states into biological signals. It is this functional homology, rather than spatial continuity, that leads us to regard them as a unified, explanatory molecular system. While MS channels serve as the key molecular units for realizing meridian function, the specific transmission of signals relies on the macroscopic anatomical and functional systems featuring distinct spatial architectures in which these units are embedded. We believe the presence of MS channels in the meridian system is not specifically tied to acupuncture, but rather serves to maintain the stability of the internal and external environments of the body. As fundamental functional structures, MS channels can transduce diverse physical stimuli, which include mechanical, electrical, acoustic, optical, magnetic, and thermal signals, into biological effects. From this perspective, it is reasonable to hypothesize that MS channels represent the fundamental biological functional units underlying meridian activity.

## 6 Application of the hypothesis to explain meridian-related issues

In recent years, significant progress has been made in the study of meridians; however, numerous challenging questions remain unresolved. The proposed hypothesis may offer a more rational framework for interpreting and addressing these issues.

### 6.1 Therapeutic effects of sham acupuncture and moxibustion

Sham acupuncture, defined as non-acupoint stimulation, often exhibits certain biological effects in clinical trials. This phenomenon challenges the traditional view asserting that therapeutic efficacy originates solely from specific acupoints. If meridian functions were entirely attributed to the unique properties of localized fixed cell populations, it would be difficult to explain why stimulating non-acupoint areas also produces physiological effects.

Based on the hypothesis that MS channels serve as the fundamental functional units of meridians, a coherent, multilevel mechanistic explanation can be provided for the efficacy of sham acupuncture. This hypothesis posits that each cell possesses a basic meridian functional unit composed of MS channels. Therefore, the effects of physical stimulation are not exclusive to acupoints but are universal among all cells that possess MS channels. The key difference between acupoints and non-acupoints lies in the distribution density and functional states of MS channels, as well as the efficiency of the local and systemic networks in which these channels are embedded.

#### 6.1.1 Universality and intensity gradient: the distribution gradient of MS channels

MS channels, such as TRPV1, TRPV4, and ASIC3, are ubiquitously present in the plasma membranes of various cell types. However, within acupoint regions that have undergone long-term evolutionary and functional specialization, the expression density and specific subtype combinations of these channels in both neuronal and non-neuronal cells, including mast cells and fibroblasts, are significantly higher than those in adjacent non-acupoint tissues (83). For example, within the subcutaneous connective tissue, muscle, and nerves at the ST36 acupoint, the expression levels of TRPV1, TRPV4, and ASIC3 are substantially higher than those in non-acupoint gluteal muscle tissue. This high-density expression enables acupoint regions to transduce identical physical stimuli more efficiently and generate stronger initial biological signals, such as Ca^2+^ influx and ATP release, thereby more readily triggering subsequent significant neuroendocrine-immune cascades. Consequently, the biological effects produced by acupoint stimulation are generally significantly stronger in intensity than those elicited by non-acupoint stimulation.

#### 6.1.2 Molecular basis for sham acupuncture efficacy: universal response of MS channels

Cells in non-acupoint regions also express MS channels, albeit potentially at lower densities or with different subtype combinations. When physical stimulation of sufficient intensity or appropriate parameters, such as electroacupuncture, pressure, or heat, is applied, the MS channels in these regions can also be activated. This activation initiates local ion fluxes, mediator release, and limited intercellular communication, such as calcium waves. This mechanism constitutes the molecular basis for the certain biological effects produced by sham acupuncture. These effects may be localized and mild, or they may produce minor systemic modulation via diffuse humoral circulation, which contrasts with the potent and specific systemic regulation induced by acupoint stimulation.

#### 6.1.3 Dynamic changes and blurring of differences: state-dependence of MS channel function

The functional activity of MS channels is not fixed but is dynamically modulated by both the intracellular and extracellular microenvironments, such as pH, inflammatory mediators, and mechanical pressure, as well as the systemic neurohumoral state, including stress hormone levels and autonomic tone. Under pathological conditions, such as chronic pain and visceral diseases, or during specific physiological states, systemic or local changes in the internal environment may extensively influence the sensitivity or expression of MS channels within cells. For instance, local tissue acidification can generally lower the activation thresholds of ASIC and TRPV1 channels. During such times, the responsiveness of cells in non-acupoint regions may be broadly upregulated, while the specific advantages of acupoint regions may be relatively diminished. This phenomenon can explain why, in certain disease models or clinical trials, the difference in therapeutic efficacy between verum and sham acupuncture sometimes becomes less significant.

In summary, the phenomenon of sham acupuncture efficacy can be coherently explained by the MS channel hypothesis. The therapeutic difference between acupoint and non-acupoint stimulation is not an absolute presence or absence of an effect, but rather a gradient of strength or weakness based on the universally present functional units, namely MS channels, in terms of their distribution density, local network efficiency, and degree of systemic integration. Acupoints serve as high-response nodes characterized by the high enrichment and functional optimization of MS channels; consequently, stimulating these regions typically produces stronger and more specific therapeutic effects. Conversely, non-acupoint regions, relying on their ubiquitously expressed MS channels, can also generate certain foundational biological effects when appropriately stimulated. This explanation unifies the seemingly contradictory traditional concepts of acupoint specificity and sham acupuncture efficacy within a single molecular mechanistic framework, thereby providing a more refined theoretical basis for the design of control groups and efficacy evaluations in clinical research.

### 6.2 Variation in meridian pathways

Propagated sensation along meridians (PSM) represents one of the core characteristics of meridian phenomena. Its path and direction are not absolutely fixed and can be altered by pathological conditions or local compression. Traditionally, some researchers have suggested that such variations may be related to volume transmission within peripheral tissues (112).Volume transmission is a novel neural regulation method that differs from traditional synaptic transmission. It refers to the diffusion of neurotransmitters or modulators through extracellular fluid to distant target cells, acting on receptors in non-synaptic regions to achieve large-scale and long-term signal regulation. Furthermore, gene expression studies have indicated that the molecular basis of PSM involves neurotransmitters, calcium ions, and cell junction pathways (113). These findings collectively point to a critical question: how is the conduction pathway of PSM formed, and why does it change? Based on the hypothesis that MS channels serve as the fundamental functional units of meridians, we propose a multilevel integrative model to explain the formation and variation of PSM pathways. This model posits that PSM is not determined by a single structure, but rather emerges as a subjective experience and functional connection that arises when the molecular activities of MS channels are guided and modulated within specific anatomical and functional systems.

#### 6.2.1 Microscopic initiation and shaping of local pathways: cluster response and environmental modulation of MS channels

PSM originates from the activation of local MS channels, such as ASIC and TRPV1, at the acupoint. These channels serve not only as direct sensors of mechanical stimulation, but their functional states are also highly dependent on modulation by the local microenvironment, which includes tissue pH and mechanical pressure, as well as neurohumoral signals. For example, a local acidic environment can lower the activation thresholds of ASIC and TRPV1 channels and alter action potential firing patterns (114), thereby affecting the intensity and pattern of the initial signals. Such alterations in channel sensitivity induced by microenvironmental variations constitute the molecular basis for the dynamic adjustment of acupoint responsiveness and local conduction pathways. Furthermore, activated MS channels trigger intercellular communication mechanisms, such as CWP, enabling the directional amplification and initial propagation of signals within the local cellular network.

#### 6.2.2 Mesoscopic conduction and pathway constraint: physical guidance of signals by anatomical carriers

Although MS channels are widely distributed, the signals they trigger do not propagate diffusely. This occurs because the cells bearing MS channels, which include sensory nerve endings, fibroblasts, and pericytes, are embedded within anatomical networks possessing specific spatial architectures. Research indicates that the interstitial spaces of fascial connective tissues, neurovascular bundles, and their surrounding low-resistance tissue fluid channels constitute the physical pathways for signal conduction. These structures function as preset tracks, guiding and constraining the propagation direction of both electrical signals and chemical signals, such as ATP, that are initiated by MS channels. When local pathological changes, such as inflammation and edema, occur, or when mechanical compression is applied, the physical properties of these anatomical channels, including flow resistance and tension, become altered. Consequently, these alterations lead to variations in the signal conduction pathways, such as detouring or shifting toward the lesion area.

#### 6.2.3 Systemic integration and central modulation: shaping pathway perception by central representation

Peripheral signals are ultimately transmitted via afferent nerves to the central nervous system. The somatosensory cortex of the brain possesses a topographic map of the body, commonly referred to as the homunculus. Furthermore, prolonged physiological or pathological stimuli, such as referred pain induced by visceral diseases, can induce plastic changes within specific neural circuits, a process known as central sensitization. Such changes may reshape the manner in which the brain perceives and integrates signals originating from specific regions of the body surface. Therefore, the subjective experience of PSM as a linear conduction, alongside the tendency of its path to deviate toward the affected area under pathological conditions, may result not only from peripheral conduction but also from the modulation exerted by the spatiotemporal integration and interpretation of these signals within the central nervous system.

In summary, the phenomenon of PSM pathway variation can be coherently explained from the perspective of the MS channel hypothesis. First, the local microenvironment and neurohumoral factors alter the initial signal characteristics by modulating MS channel sensitivity. Subsequently, these signals are guided along specific anatomical carrier networks, the physical states of which are altered by pathological conditions or mechanical compression. Finally, the signal conduction pathway is further shaped by the integration and perception processes of the central nervous system, which are based on past experiences and current states. Therefore, the specific pathway of a meridian does not represent a static anatomical conduit, but rather serves as a functional manifestation of the dynamic interaction between the molecular activities of MS channels and the multilevel physiological systems of the body, which include the local microenvironment, anatomical networks, and central integration. This integrative model unifies traditional volume transmission concepts and gene expression findings within a testable mechanistic framework spanning from the molecular level to systemic levels.

### 6.3 Meridians and transplantation

The success of organ transplantation poses a fundamental challenge to traditional meridian theory. Specifically, when the neural, vascular, and connective tissue connections of a transplanted organ, such as a kidney, are completely severed through procedures like denervation and the use of artificial grafts, how can the recipient’s meridians influence the function of the transplanted organ across this anatomical discontinuity? TCM theory posits that meridians dictate life and death, implying that an allograft lacking meridian regulation would function abnormally. However, clinical practice demonstrates that such denervated transplanted kidneys, which rely solely on blood supply, can still survive and function. This contradiction suggests that the regulatory influence of meridians on organs may not depend on continuous macroscopic anatomical structures, but rather operates through more fundamental biological mechanisms.

Based on the hypothesis that MS channels serve as the fundamental functional units of meridians, a coherent explanatory framework grounded in modern biology can be provided for this challenge. This hypothesis posits that every cell, including those of the transplanted organ, possesses a basic meridian functional unit composed of MS channels. The influence of the recipient’s meridians on the transplanted organ is not exerted through direct neurovascular channel connections, but rather through systemic physical and humoral signals, which include blood pressure, shear stress, osmotic pressure, circulating hormones, and cytokines, to regulate MS channels on the cell membranes of the transplanted organ, thereby achieving remote functional coupling. This perspective is corroborated by findings from renal developmental biology. Research demonstrates that the regulatory influence of renal nerves on the kidney exhibits distinct stages. Experiments indicate that during the rapid developmental phase prior to postnatal days 12 to 14 in rabbits, renal denervation significantly hinders kidney growth and the maturation of nephron structures. Conversely, after 18–30 days of age, when the kidney is essentially mature, denervation exerts no significant effect on kidney weight or structure (115). This suggests that following organ maturation, the direct trophic regulatory effects of nerves on morphological structures diminish, and the maintenance of organ function may rely more heavily on other mechanisms, such as the continuous response of MS channels to systemic physical and chemical signals. Importantly, MS channels themselves may have already played a critical role during kidney embryogenesis by participating in nephron differentiation and tissue organization. Therefore, the inherent MS channel network on the cells of the transplanted kidney may not only carry the potential for functional regulation in adulthood but may also represent a form of molecular memory or a functional blueprint established during kidney development that is linked to specific functions. When the recipient regulates these channels via systemic signals, the recipient essentially couples with a fundamental functional system deeply embedded in the developmental history of the organ. However, denervation is not without consequences. Studies indicate that denervated kidneys are prone to fibrosis, which may be related to an imbalance in local mechanical signaling homeostasis following the loss of neural regulation, leading to the aberrant activation of profibrotic MS channel pathways, such as the Piezo1 pathway (116).

#### 6.3.1 Re-examining clinical phenomena and the concept of meridian transplantation

Clinical studies have explored the therapeutic effects of acupuncture on patients with chronic renal allograft nephropathy. The patients were categorized into groups receiving acupoint stimulation along different meridians. The results demonstrated that stimulating acupoints along the Spleen Meridian significantly reduced serum creatinine and 24-h urine protein levels compared to pretreatment baseline levels, yielding superior outcomes compared to either Kidney Meridian stimulation or the control groups (117). This finding suggests that the transplanted kidney may have established a new functional connection with the recipient’s Spleen Meridian, rather than simply reconnecting with its original Kidney Meridian. This phenomenon has been termed meridian transplantation, but its underlying mechanism remains unclear.

#### 6.3.2 MS channel hypothesis: a mechanism for functional coupling across anatomical discontinuity

In cases of denervated kidney transplantation utilizing artificial grafts, the transplanted kidney lacks direct neural and connective tissue continuity with the recipient. Our hypothesis proposes that the inherent MS channels on the cells of the transplanted kidney, which include TRPV4, Piezo1, and epithelial sodium channels (ENaC), constitute its intrinsic meridian functional units. The meridians of the recipient can influence these channels via two primary pathways. First, regarding mechanical signals, changes in the blood pressure and hemodynamics of the recipient generate vessel wall shear stress and circumferential stress. These mechanical forces can directly act on MS channels, such as TRPV4 and Piezo1, located on the vascular endothelial cells and tubular cells of the transplanted kidney via blood circulation. Second, concerning humoral signals, hormones, cytokines, and other chemical substances released by the neuroendocrine system of the recipient reach the transplanted kidney via humoral circulation, thereby modulating the functional states or expression levels of MS channels on its cells. ASICs, K2Ps, and TRPs represent three types of channels that are strongly and widely influenced by chemical factors, serving as mechanosensitive-multimodal sensors themselves. The ENaC/DEG complex is primarily regulated by chemical factors, including ions and hormones, although its mechanosensitivity has been reported in certain subtypes. Piezo channels function as the purest mechanosensors, given that their activation clearly depends on mechanical forces. However, their functions can be indirectly modulated by chemical factors, such as the membrane lipid environment.

Studies have demonstrated that mechanosensing is crucial for kidney function. For instance, TRPV4 may act as an osmoreceptor within the kidney, participating in the regulation of sodium and water balance. Furthermore, by sensing pressure changes in the afferent glomerular arteriole, TRPV4 mediates calcium influx, thereby inhibiting renin secretion and contributing to the negative feedback regulation of blood pressure (118). The acute downregulation of ENaC and aquaporin-2 (AQP2) following kidney transplantation is considered a protective adaptation to reduce energy consumption within the nephron (119). These processes either depend on or regulate the MS channels located on kidney cells.

#### 6.3.3 A new perspective on the spleen meridian connection

Why does the Spleen Meridian, rather than the Kidney Meridian, exhibit a stronger functional association with the transplanted kidney? Our hypothesis offers a speculative but plausible explanation: the traditional functions of the Spleen Meridian involve transporting and transforming fluids, as well as governing Qi and blood, which correspond to the modern physiological concepts of fluid balance, immune regulation, and blood component management. The survival and function of a transplanted kidney are highly dependent on the systemic hemodynamic stability, immune tolerance status, and internal environmental homeostasis of the recipient. Stimulating Spleen Meridian acupoints may more effectively optimize these systemic conditions, such as improving microcirculation and modulating inflammatory cytokines, by regulating the autonomic neuroendocrine-immune network of the recipient, thereby creating a more favorable functional niche for the transplanted kidney cells via their MS channels. In other words, by regulating the overall homeostasis of the recipient, the Spleen Meridian may indirectly yet potently influence the microenvironment and function of MS channels located on transplanted kidney cells, thereby establishing a dominant functional coupling.

In summary, the issue of meridian connection in transplanted organs can be newly understood through the MS channel hypothesis. The influence of the meridians of the recipient on the transplanted organ is not achieved by transplanting a tangible channel, but rather by remotely regulating the inherent fundamental functional units composed of MS channels within the cells of the transplanted organ. This regulation occurs via systemic physical signals, such as mechanical forces, and chemical signals, such as humoral factors, from the recipient, thereby achieving functional integration. The clinically observed specific connection between the transplanted kidney and the Spleen Meridian may reflect the critical role of the Spleen Meridian in regulating systemic homeostasis, thereby providing the optimal remote regulatory background for the MS channel network of the transplanted kidney. This explanation transforms the traditional concept of meridian transplantation into a testable mechanistic model based on modern cellular biology, molecular biology, and systems physiology, offering a new theoretical perspective for understanding the integrative therapeutic effects of TCM and Western medicine following organ transplantation.

## 7 Conclusion and future prospects

Through an analysis of the biological and biophysical properties of meridians and MS channels, particularly their critical role in mediating the therapeutic effects of acupuncture, we propose the following hypothesis: MS channels represent the fundamental biological functional units of meridians. Specifically, each cell may possess a basic meridian functional unit composed of MS channels. Similar to other structures involved in physical signal transduction, these channels constitute essential tissue components of the meridian system. MS channels do not represent continuous anatomical pathways connecting distant organs; rather, they act as local initiators at acupoints, integrators within the nervous system, and effector sensors within visceral effector organs. These channels serve as the molecular starting points of a multilevel cascade, rather than the conduits themselves; meanwhile, classical neural and humoral pathways act as the crucial mediating systems for signal propagation from acupoints to visceral effectors.

This hypothesis posits that the local signal initiation and amplification provided by MS channels are necessary conditions for realizing meridian functions. However, how these discrete molecular events are ultimately integrated into functional pathways with specific surface-viscera correspondences that can be perceived and regulated, known as meridians, through the topographic projections of the nervous system, the circulation of the humoral system, and the mechanical coupling of the connective tissue network remains a key mechanism to be elucidated. Furthermore, this integration represents a core prediction of this hypothesis that requires future research validation.

This hypothesis offers a coherent theoretical framework for interpreting numerous meridian-related phenomena and is expected to deepen the understanding of the biological functions of meridians. Concurrently, it raises several important research questions. For instance, how do the types, quantities, and spatial distributions of MS channels relate to their functional roles within meridians? Do distinct meridians arise from specific types or combinations of MS channels? Are the MS channels located along different meridians functionally linked to particular visceral effector cells? Furthermore, how can the role of MS channels within meridians be investigated through mechanotransduction modeling, bioelectrical signal networks, and systems-level physiology? These open questions provide a basis for further mechanistic studies and are likely to stimulate deeper investigation into the biology of MS channels. Nevertheless, validating the hypothesis that MS channels serve as the fundamental biological units of meridians will require extensive and sustained research efforts.

In summary, this hypothesis proposes that MS channels serve as the fundamental functional units of meridians. The role of these units spans the entire lifespan. During the embryonic period, they function as potential architects for organ morphogenesis and the establishment of specific surface-viscera connections. In adulthood, they transform into regulators that mediate physical stimuli, maintain internal environmental homeostasis, and realize the modulatory effects of acupuncture. This dual role, spanning development and adulthood, closely integrates the traditional functional descriptions of meridians with modern developmental biology, cellular biology, and molecular biology, thereby providing a unified framework spanning from origin to function for understanding the biological essence of meridians.
